# Alexithymia and the Reduced Ability to Represent the Value of Aversively Motivated Actions

**DOI:** 10.3389/fpsyg.2018.02587

**Published:** 2018-12-18

**Authors:** Francesca Starita, Giuseppe di Pellegrino

**Affiliations:** Center for Studies and Research in Cognitive Neuroscience, Department of Psychology, University of Bologna, Cesena, Italy

**Keywords:** alexithymia, instrumental learning, reinforcement learning, action value, probabilistic selection task

## Abstract

Alexithymia is a subclinical trait defined by difficulties in identifying and describing feelings and a cognitive style avoidant of introspection. Extensive literature shows that alexithymia is characterized by multifaceted impairments in processing emotional stimuli. Nevertheless, the mechanisms that may account for such impairments remain elusive. Here, we hypothesize that alexithymia may be understood as impairment in learning the emotional value of one’s own actions and test this comparing performance of participants with high (HA) and low (LA) levels of alexithymia on a probabilistic selection task. Results show that, compared to LA, HA need more time to learn the value of individual stimuli and associated actions as difference in reinforcement rate between stimuli decreases. In addition, HA appear less able to generalize the value of previously learned actions that lead to a negative outcome, to make adaptive choices in a new context, requiring more time to avoid the most negative stimulus between two negative stimuli. Together, the results indicate that individuals with alexithymia show impaired learning of the value of aversively motivated actions. We argue that this impairment may hinder the construction of internal representations of emotional stimuli and actions and represent a mechanism that may account for the difficulties of alexithymia in processing emotional stimuli.

## Introduction

Alexithymia is a subclinical trait defined by difficulties in identifying feelings and describing them to others, and a style of thinking more focused on the concrete aspects of life rather than on introspection (Sifneos, 1973; Taylor et al., 1991). Individuals with alexithymia represent about 10% of the general population (Taylor et al., 1991) and show multifaceted impairments in processing emotional stimuli. For example, they have impairments in the identification of emotional stimuli (Grynberg et al., 2012; Ihme et al., 2014a,b; Starita et al., 2018), the physiological response to those stimuli (Franz et al., 2003; Neumann et al., 2004; Pollatos et al., 2008; Bermond et al., 2010), the regulation of such response (Swart et al., 2009; Pollatos and Gramann, 2012) and its use to effectively guide decision making (Ferguson et al., 2009; Patil and Silani, 2014a,b; Scarpazza et al., 2017). Crucially, despite this evidence, the mechanisms that may account for such difficulties remain poorly understood.

Cognitive theories of emotional experience argue that the subjective experience of emotion is a higher-order cognitive interpretation of lower-order information, coming from within the body and the external environment (Schachter and Singer, 1962; LeDoux, 1998; Craig, 2003, 2009; Barrett et al., 2007; Barrett, 2017a,b; LeDoux and Brown, 2017; LeDoux and Hofmann, 2018). In line with this, a recent account of alexithymia proposes that impairments in the accurate perception of the physiological signals from the body, i.e., interoception, even in absence of emotional stimuli, are core to alexithymia (Bird and Viding, 2014). This account is supported by evidence of reduced interoception in the cardiac domain (Brewer et al., 2015; Murphy et al., 2018a) as well as respiratory, muscular effort and taste (Murphy et al., 2018b). Additionally, in the wake of the theory of embodied emotion, which poses the accent on the need for a somatovisceral and motor response to the presentation of emotional stimuli to effectively experience emotions (Niedenthal, 2007; Niedenthal et al., 2009), another account of alexithymia has been proposed. According to this (Scarpazza and di Pellegrino, 2018), alexithymia would be characterized by a failure in emotional embodiment, as evidenced by impaired mimicry (Sonnby-Borgström, 2009; Scarpazza et al., 2018) and aberrant visual remapping of touch when viewing emotional facial expressions (Scarpazza et al., 2014, 2015).

Crucially, which stimuli have affective value in the first place is the result of a learning process (Pavlov, 1927; LeDoux, 1998, 2000, 2012; Barrett, 2017a,b; LeDoux and Brown, 2017). In fact, only a restricted range of stimuli is biologically programmed to trigger an emotional response, i.e., appetitive and aversive unconditioned stimuli. Organisms actively construct the internal representations of emotional stimuli, in order to include, alongside unconditioned stimuli, those associated with them, through a process of emotional learning. These internal representations are predictive models that enable individuals to anticipate the emotional future, so that organisms can appropriately prepare to respond to coming emotional stimuli, rather than simply react to them once they have occurred (Öhman and Mineka, 2001; McNally and Westbrook, 2006; den Ouden et al., 2012). These predictive representations are not only fundamental for effective recognition, response and response regulation to the emotional stimuli *per se*, but also for anticipating the consequences of these stimuli enabling optimal decision making (Bubic et al., 2010), processes that are all impaired in alexithymia.

In line with this, we have previously proposed a different account of alexithymia and argued that alexithymia may be understood as impairment in effectively learning the emotional value of encountered stimuli, and showed that individuals with alexithymia have reduced psychophysiological response to aversively conditioned stimuli during Pavlovian threat conditioning, despite preserved response to unconditioned stimuli (Starita et al., 2016). Therefore, individuals with alexithymia appear able to respond to stimuli, which are biologically prepared to trigger an emotional response. Nevertheless, they appear unable to use such information to construct an internal representation of emotional stimuli that includes, alongside stimuli that unconditionally elicit an emotional response, those that are associated with them. Here, we extend this investigation and ask whether such difficulty is present also when having to learn the value of one’s actions. In fact, in everyday life, the organism is an active agent in its surrounding environment, changing its behavior based on the outcome it might lead to, in order to select those actions that can increase survival. Specifically, through a process named instrumental learning, organisms learn to attribute an affective value to previously neutral actions depending on the outcome they lead to so that actions leading to reward will be repeated, while action leading to punishment will be terminated (Daw and Tobler, 2014). In addition, the environment is ever-changing, so that the same stimuli are rarely encountered in the same context twice. Therefore, organisms are also required to exhibit adaptive behavior, when the same stimuli are encountered in a novel context. To ensure this, the information learned about the value of actions associated to previously encountered stimuli has to be generalized to novel contexts.

**FIGURE 1:**
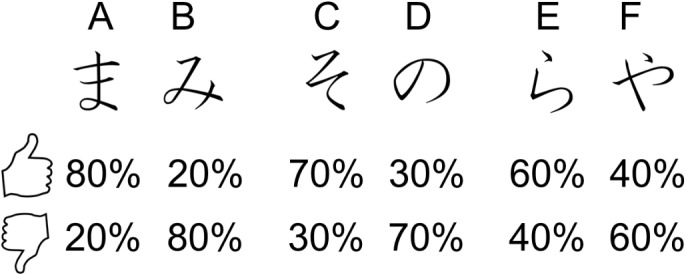
Illustration of examples of stimuli and the feedback probability associated with each stimulus for each type of feedback.

Given the above information, the aim of the current study is to investigate whether individuals with alexithymia show impairments in learning the value of their actions and in using this information effectively to ensure adaptive behavior in novel contexts. To this end, individuals with low (LA) and high (HA) levels of alexithymia as measured on the Toronto Alexithymia Scale (Taylor et al., 2003) were recruited to participate. Therefore, our investigation concerns individuals with type II alexithymia, characterized by preserved emotionality but poorly developed emotional cognition, rather than individuals with type I or affective alexithymia, characterized by blunted emotionality together with poorly developed emotional cognition (as defined by Bermond, 1997). Participants completed a modified version of the Probabilistic Selection Task (PST) (Frank et al., 2004, 2005). The PST includes two phases: learning and testing. During the learning phase, participants complete an instrumental learning task, which includes three pairs of stimuli (AB, CD, and EF). Within each pair, choosing one stimulus is more likely to lead to reward (and less likely to lead to punishment) than choosing the other. Importantly, the probability of reward and punishment differs for each stimulus (Figure 1), so that each stimulus and the choice associated to it acquire a more or less positive or negative value compared to the remaining ones. On each trial, participants choose one stimulus of the pair and reward (positive feedback) or punishment (negative feedback) following the choice is provided. By trial and error, participants are required to learn the stimulus in each pair more likely to lead to reward. Then, during testing, participants are again faced with pairs of stimuli; however, all possible combinations of the stimuli encountered during learning are presented. Participants’ task remains to choose the stimulus in each pair more likely to lead to reward; nevertheless, no feedback is provided about the choice. The testing phase enables to assess whether participants learned more from reward or punishment and whether they are able to generalize to a new context the information previously learned about the value of each stimulus and the choice associated to it, to make effective choices when the old stimuli are presented within new pairs. If alexithymia is indeed related to impairments in learning the value of one’s actions and in generalizing the acquired learning to new contexts, we expect HA to show worse performance than LA both in the learning and testing phase of the PST.

## Materials and Methods

### Participants

Three-hundred individuals completed the 20-item Toronto Alexithymia Scale (TAS-20; Taylor et al., 2003). Depending on the score, individuals were classified as LA (TAS-20 ≤ 36, *n* = 80) or HA (TAS-20 ≥ 61, *n* = 46) (Franz et al., 2004). Individuals from these two groups were then randomly contacted to participate in the study, until the *a priori* target for sample size was reached. Specifically, *a priori* targets for sample size and data collection stopping rule were based on sample and effect sizes reported in the literature on the PST (e.g., Chase et al., 2010; Cavanagh et al., 2011).

Once in the laboratory, the alexithymia module of the structured interview for the Diagnostic Criteria for Psychosomatic Research (DCPR; Mangelli et al., 2006) was administered to increase reliability of screening and confirm TAS-20 classification (LA: DCPR < 3, HA: DCPR ≥ 3). Participants with discordant classification on the two measures did not complete the task (*n* = 8). Due to the high co-occurrence of alexithymia and depression (Li et al., 2015), participants completed the Beck Depression Inventory (Beck et al., 1961) and did not complete the experimental task in case their score was higher than the moderate/severe depression cut-off (i.e., 19, *n* = 3). Additionally, due to the high prevalence of alexithymia in clinical populations, such as populations with anxiety (Berthoz et al., 1999), eating (Panaite and Bylsma, 2012) and addiction disorders (Farges et al., 2004; Kun and Demetrovics, 2010; Craparo et al., 2016) only volunteers with no history of major medical, neurological or psychiatric disorders (self-reported) were included.

Forty-one participants completed the study: 20 LA (six males; age *M* = 21.44, *SD* = 1.65 years; TAS-20 *M* = 31.89, *SD* = 2.58); 21 HA (six males; age *M* = 21.83 *SD* = 1.85 years; TAS-20 *M* = 64.70, *SD* = 4.59). All participants had equivalent educational backgrounds and were students at the University of Bologna. The study was designed and conducted in accordance with the ethical principles of the World Medical Association Declaration of Helsinki and was approved by the Bioethics Committee of the University of Bologna. All participants gave informed written consent to participation after being informed about the procedure of the study.

### Independent Measures

The experimental task consisted in a modified version of the PST (Frank et al., 2004, 2005). This includes two phases: learning and testing.

#### Learning

This phase was a reinforcement learning procedure. On each trial a pair of stimuli consisting of hiragana characters appeared on the screen. Every time a pair appeared, the participant chose one of the two stimuli pressing a key on the keyboard. Following the choice, feedback appeared on the screen indicating whether the choice was correct (reward) or incorrect (punishment). These consisted of a hand with a thumb up or down, respectively. In total, there were three pairs of stimuli (AB, CD, and EF). In each pair, each stimulus had a predetermined probability of being followed by the correct feedback. Specifically, for the AB pair, choosing A led to correct feedback (reward) 80% of the time and incorrect (punishment) in the remaining 20% of the time, whereas B led to correct feedback (reward) only 20% of the time. For the CD pair, choosing C led to correct feedback (reward) 70% of the time, whereas D led to correct feedback (reward) only 30% of the time. For the EF pair, choosing E led to correct feedback (reward) 60% of the time, whereas F led to correct feedback (reward) only 40% of the time (Figure 1). Participants’ task was to learn to choose the stimulus in each pair that leads to correct feedback in the majority of trials.

A performance criterion was introduced for each pair to ensure participants achieved comparable level of learning before moving to the testing phase. Specifically, this was 65% of A for AB, 60% of C in CD and 55% of E in EF. Learning was evaluated at the end of each training block consisting of 60 trials (20 per stimulus pair) for a maximum of four blocks. Participants who did not achieve the criterion after four blocks were excluded from further analysis. After achieving the criterion, participants proceeded to the testing phase.

**FIGURE 2:**
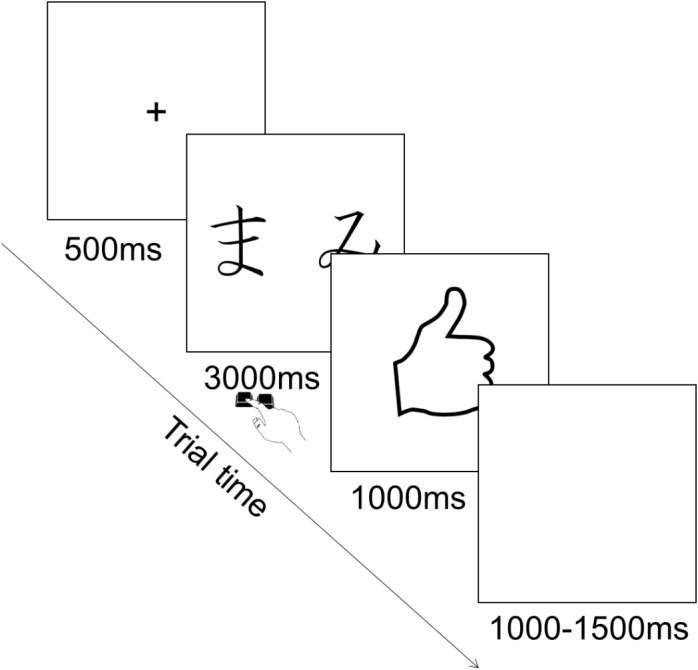
Illustration of experimental trial for the learning phase.

Each trial consisted in the presentation of a fixation cross in the center of the screen for 500 ms, followed by the presentation of the pair of stimuli during which participants could provide their choice by pressing the corresponding key. Key press terminated stimulus presentation and participants had a maximum of 3000 ms to provide their answer. This was followed by the feedback for 1000 ms, followed by an inter trial interval of 1000–1500 ms during which a blank screen was presented (Figure 2). The order of presentation of stimuli was randomized across trials. The type of stimuli constituting each pair was counterbalanced among participants.

#### Testing

This phase enabled to evaluate how the acquired learning affected choice behavior when the same stimuli are presented in a new context. So, the old pairs of stimuli were presented in addition to new pairs of stimuli resulting from all the possible combinations of pairs of stimuli.

On each trial a pair appeared on the screen and participants chose one of the two stimuli. No feedback was given about the choice. Participants’ task was to choose the stimulus in each pair they thought was the correct one based on what they had learned in the previous phase. Participants were also told to guess when they were not sure about which stimulus to choose.

**FIGURE 3:**
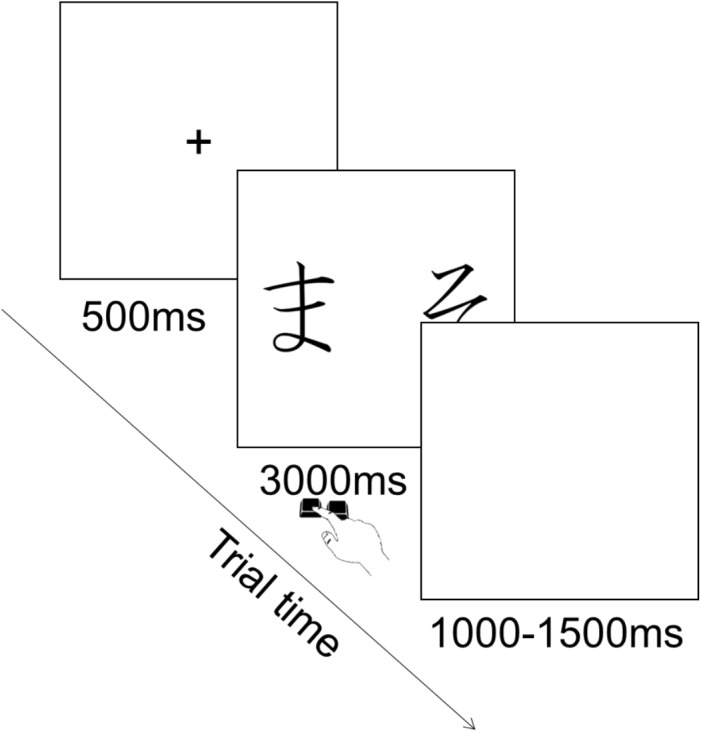
Illustration of experimental trial for the testing phase.

Each trial consisted in the presentation of a fixation cross in the center of the screen for 500 ms, followed by the presentation of the pair of stimuli during which participants could provide their choice by pressing the corresponding key. Key press terminated stimulus presentation and participants had a maximum of 3000 ms to provide their answer. This was followed by an inter trial interval of 1000–1500 ms during which a blank screen was presented (Figure 3). The order of presentation of stimuli was randomized across trials. There were 90 trials in total (six per pair).

### Dependent Measures

The following dependent measures were computed from the data collected during the learning phase.

#### Number of Blocks Completed During Learning

The number of blocks completed in order to achieve the performance criterion was counted for each participant to then test whether there were any group differences.

#### Early Learning

Considering that all participants completed at least one block of learning, the percentage of accurate response and average response times for accurate responses for the first block were evaluated to test differences between groups in early acquisition of learning (Waltz et al., 2007).

#### Degree of Exploration During Early Learning

The probability of changing response following either positive or negative feedback was calculated during the first block of learning in order to test any group differences.

The following dependent measures were computed from the data collected during the testing phase.

#### Retention of Learning

We verified that subjects retained the performance criterion for successful learning also during the testing phase, to ensure learning was retained even when actions were no more reinforced by feedback. Consequently, participants whose accuracy in choosing the correct stimulus when faced with old pairs (AB, CD, or EF) did not equal or exceed the performance criterion were excluded from further analysis because their data were not interpretable. Then differences between groups in accuracy and response times for the old pairs were tested.

#### Generalization of Learning to a Novel Context

On each trial, participants were faced with one out of four possible types of choice (Table 1). First, they could be faced by a pair consisting of one correct stimulus and one incorrect stimulus (conflict of choice: low conflict), and where the probability of the correct stimulus of having been rewarded was greater than the probability of the incorrect stimulus of having been punished (type of choice: choose positive). This included AD, AF, and CF pairs. Second, they could be faced by a pair consisting of one correct stimulus and one incorrect stimulus (conflict of choice: low conflict) and where the probability of the incorrect stimulus of having been punished was greater than the probability of the correct stimulus of having been rewarded (type of choice: avoid negative). This included BC, BE, and DE pairs. Third, they could be faced by a pair consisting of two correct stimuli (conflict of choice: high conflict) and where one had higher probability of having been previously rewarded compared to the other (type of choice: choose positive). This included AC, AE, and CE pairs. Four, they could be faced by a pair consisting of two incorrect stimuli (conflict of choice: high conflict) and where one had higher probability of having been previously punished compared to the other (type of choice: avoid negative). This included BD, BF, and DF pairs. The percentage of accurate response and the average response time for accurate choices for each participant and for each type of choice were calculated to test differences in performance between groups during testing.

**Table 1:** Illustration of the different types of choice during the testing phase.

Stimulus	Value of stimuli	Conflict of choice	Type of choice
AC	A: 80% positive C: 70% positive	High	Choose positive
AD	A: 80% positive D: 70% negative	Low	Choose positive
AE	A: 80% positive E: 60% positive	High	Choose positive
AF	A: 80% positive F: 60% negative	Low	Choose positive
BC	B: 80% negative C: 70% positive	Low	Avoid negative
BD	B: 80% negative D: 70% negative	High	Avoid negative
BE	B: 80% negative E: 60% positive	Low	Avoid negative
BF	B: 80% negative F: 60% negative	High	Avoid negative
CE	C: 70% positive E: 60% positive	High	Choose positive
CF	C: 70% positive F: 60% negative	Low	Choose positive
DE	D: 70% negative E: 60% positive	Low	Avoid negative
DF	D: 70% negative F: 60% negative	High	Avoid negative

## Results

### Alexithymia Groups Did Not Differ Significantly in the Number of Blocks Required to Complete Learning

First, we tested group differences in the number of blocks required to achieve the performance criterion. One LA and one HA were excluded from this and further analysis because they failed to achieve the performance criterion after four blocks of training. An independent sample *t*-test showed no significant difference between LA and HA in the average number of blocks completed to achieve the performance criterion [*t*(37) = 0.73, *p* = 0.467; *M*_LA_ = 1.78, *M*_HA_ = 1.60]. The groups required a comparable number of blocks to achieve the performance criterion.

### Alexithymia Groups Did Not Differ Significantly in the Degree of Exploration During Early Learning

Then, we tested group differences in the degree of exploration during the first block of learning. A 3 × 2 × 2 RM ANOVA (type of pair: AB, CD, EF; type of feedback: correct, incorrect; group: LA, HA) showed a significant main effect of type of feedback [*F*(1,37) = 36.97, *p* < 0.001, partial η^2^ = 0.50]. *Post hoc* comparison indicated that on any given trial, participants were more likely to switch choice toward the other stimulus in the pair, if they had received incorrect feedback in the previous trial of the same pair than if they had received correct feedback (*p* < 0.001, *M*_correct_ = 0.17, *M*_incorrect_ = 0.31). All other effects were not significant (all *p*s ≥ 0.125) and in particular, there was no significant main effect or interaction with the factor group indicating that groups had comparable degree of exploration while learning the correct stimulus in each pair.

### HA Require More Time to Choose the Correct Stimulus in EF Than AB Pairs

Then, we tested group differences in accuracy and response times when learning to choose the correct stimulus during the first block of learning. A 3 × 2 RM ANOVA on the accuracy (type of pair: AB, CD, EF; group: LA, HA) showed a main effect of the type of pair [*F*(2,74) = 5.40, *p* = 0.006, partial η^2^ = 0.12]. *Post hoc* comparison indicated that at the end of the first block participants achieved lower response accuracy to the EF (*M* = 0.640) pair compared to AB (*M* = 0.785; *p* = 0.009) and CD (*M* = 0.770; *p* = 0.004), while there was no significant difference in the accuracy between the response to AB and CD (*p* = 0.773). In addition, there was no main effect or interaction with the factor group (all *p*s ≥ 0.868).

**FIGURE 4:**
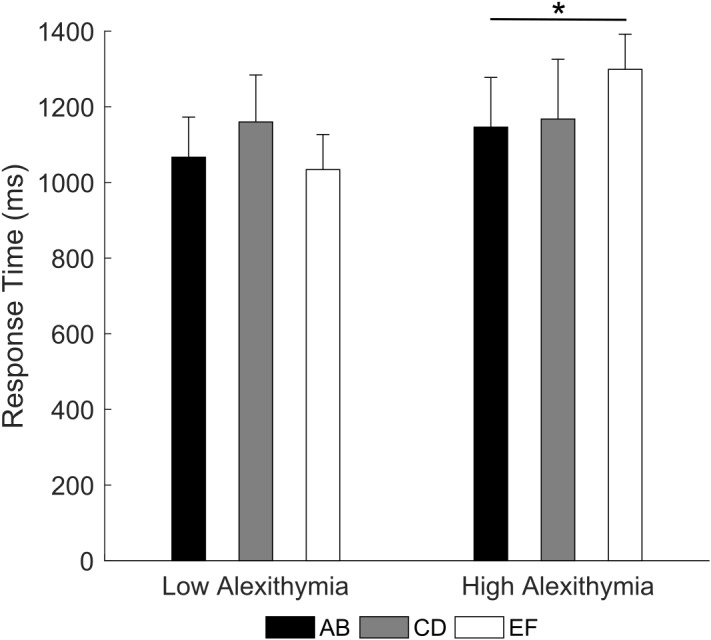
Mean response time for each stimulus pair as a function of alexithymia group. In the high alexithymia group, participants were slower in choosing the correct stimulus in the EF than in the AB pair. Error bars represent standard errors. Significant differences are indicated as follows: ^∗^*p* < 0.05.

Crucially, the 3 × 2 RM ANOVA (type of pair: AB, CD, EF; group: LA, HA) on response times for correct trials (note that only participants with at least one accurate response on any pair were included) showed a significant pair by group interaction [*F*(2,70) = 3.45, *p* = 0.004, partial η^2^ = 0.09; Figure 4]. *Post hoc* comparison indicated that LA had no significant difference in response times between the three pairs (all *p*s ≥ 0.069), on the contrary, HA were slower when choosing the correct stimulus in the EF pair (*M* = 1299.1 ms) than in the AB pair (*M* = 1146.3 ms, *p* = 0.035). The main effects were not significant (all *p*s ≥ 0.255). These results indicate that as the difference in reinforcement rate between two stimuli decreases, HA may find increasingly difficult to learn the value of individual stimuli and associated actions, requiring more time to maintain choice accuracy.

### Alexithymia Groups Did Not Differ Significantly in Retention of Learning

Then, we tested group differences in retention of learning (i.e., maintain performance criterion on old stimuli pairs) during the testing phase. Two LA and three HA were excluded from this and further analysis because they failed to retain the acquired learning. Among participants who retained learning, a 3 × 2 RM ANOVA on the accuracy (type of pair: AB, CD, EF; group: LA, HA) showed no significant main effect or interaction (all *p*s ≥ 0.503). Similarly, also the 3 × 2 RM ANOVA on the response times, showed no significant main effect or interaction (all *p*s ≥ 0.239). These results indicate that groups had comparable retention of learning about the value of stimuli and associated actions during the testing phase.

### HA Require More Time to Avoid a Negative Stimulus Than Choosing a Positive One, When in a Novel High-Conflict Context

Finally, we tested group differences in the ability to generalize the learned value of stimuli and associated actions, when facing new pairs of stimuli. The 2 × 2 × 2 RM ANOVA (type of learning: choose positive, avoid negative; type of conflict: low conflict, high conflict; group: LA, HA) on mean accuracy showed a significant main effect of conflict [*F*(1,32) = 91.59, *p* < 0.001, partial η^2^ = 0.74]. *Post hoc* comparisons showed that participants were less accurate when facing pairs with high conflict choice (*M* = 0.91) than low conflict choice (*M* = 0.54; *p* < 0.001). No other main effects and interactions were significant (all *p*s ≥ 0.208).

**FIGURE 5:**
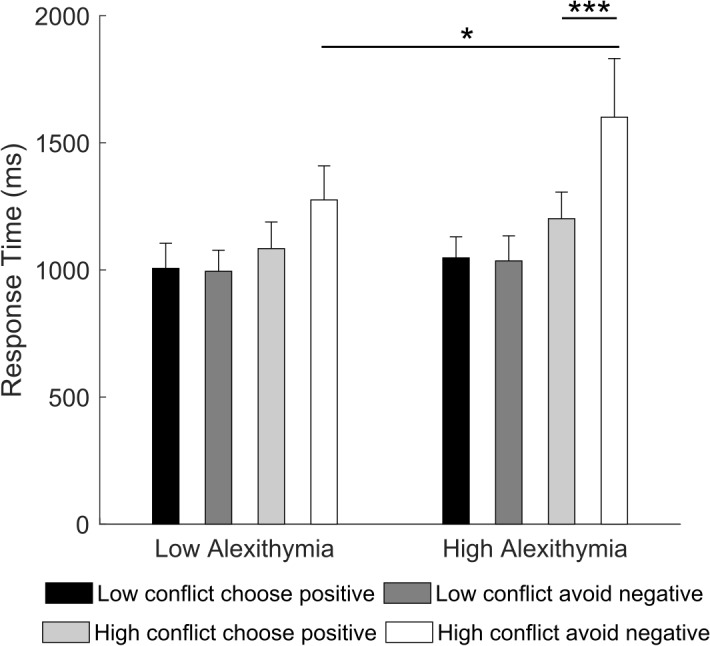
Mean response time for “choose positive” and “avoid negative” trials in the “low conflict” and “high conflict” conditions as a function of alexithymia group. In the high alexithymia group, participants were slower when avoiding the negative than when choosing the positive stimulus in the high conflict condition. Error bars represent standard errors. Significant differences are indicated as follows: ^∗^*p* < 0.05, ^∗∗∗^*p* < 0.001.

Crucially, differences between LA and HA became evident in the 2 × 2 × 2 RM ANOVA (type of learning: choose positive, avoid negative; type of conflict: low conflict, high conflict; group: LA, HA) on the reaction times. Results showed a significant main effect of type of learning [*F*(1,32) = 9.18, *p* = 0.005, partial η^2^ = 0.22], type of conflict [*F*(1,32) = 15.76, *p* < 0.001, partial η^2^ = 0.33] and type of learning by type of conflict interaction [*F*(1,32) = 15.45, *p* < 0.001, partial η^2^ = 0.32]. However, these were all qualified by a significant type of learning by type of conflict by group interaction [*F*(1,32) = 5.09, *p* = 0.031, partial η^2^ = 0.14]. *Post hoc* comparisons showed that in high conflict trials, HA were slower when accurately avoiding a negative stimulus (*M*_negative_ = 1784.2 ms) than when choosing a positive one (*M*_positive_ = 1245.3, *p* < 0.001) and they were slower than LA (*M*_negative_ = 1189.3 ms, *p* = 0.016; Figure 5). No significant within or between group effects were found for low conflict trials (all *p*s ≥ 0.571). This result suggests that, in novel contexts of low conflict, participants require comparable amount of time to choose positive or avoid negative stimuli. On the contrary, in high conflict, HA require more time to avoid a negative stimulus compared to choosing a positive one as well as more time than LA. LA, instead, require comparable amount of time to make either type of choice.

To better understand which of the three components of alexithymia influenced the current results, we ran a stepwise multiple regression using participants’ scores on the TAS-20 subscales [i.e., difficulty in identifying feelings (DIF), difficulty in describing feelings (DDF), and externally oriented thinking (EOT)] as independent variables and average response time on “avoid negative” trials as dependent variable. We found that only the score on the externally oriented thinking subscale made a significant contribution to the regression [R^2^ = 0.16, F(1,32) = 5.99, p = 0.020; EOT: β = 0.40, t(32) = 2.45, p = 0.020; DIF: p = 0.747; DDF: p = 0.290]. This result indicates that the more participants had a concrete cognitive style, the slower they were at avoiding negative stimuli in high conflict novel context.

## Discussion

In the current study, participants with low (LA) and high (HA) levels of alexithymia completed a modified version of the Probabilistic Selction task (PST; Frank et al., 2004, 2005), in order to test whether individuals with alexithymia show impairments in learning the value of their actions and in generalizing such learning to make adaptive choices in a novel context. When examining performance accuracy, the results were in line with the previous literature. During learning, participants were more likely to change choice of stimulus in a pair if their previous choice received incorrect than if it received correct feedback (Frank et al., 2007). This indicates that the two types of feedback were effective as punishment and reward: participants repeated rewarded choices and terminated punished ones. Interestingly, this trial-by-trial adjustments in behavior have been argued to reflect the ability to maintain positive and negative outcomes in working memory, rather than sensitivity to reward and punishments *per se* (Frank et al., 2007; Frank and Kong, 2008) and results suggest that this was not affected in alexithymia. During testing, when faced with the new pairs of stimuli, participants showed no difference between choosing positive and avoiding negative stimuli (replicating Frank et al., 2007, 2005), but they were less accurate in making the correct choice in high conflict than in low conflict situations. Indeed, in a novel context, having to choose between one positive and one negative stimulus seems easier that having to choose between two positive or two negative stimuli, which differ only in their reinforcement rate.

Crucially, HA showed significant impairments in performance during both learning and testing. During learning, the two groups did not differ significantly in the number of blocks completed to learn the value of stimuli, the degree of exploration following reward or punishment, and the accuracy in identifying the correct stimulus in each pair. Nevertheless, analysis on reaction time, showed that HA, but not LA, needed more time, during the early phase of learning, to identifying the correct stimulus in the EF than in the AB pair. Importantly, while in the AB pair, the percentage difference in reinforcement rate between stimuli was 60%, in the EF pair, the percentage difference in reinforcement rate between stimuli was only 20%. Therefore, as difference in reinforcement rate between two stimuli decreases, HA may find increasingly difficult to learn the value of individual stimuli and associated actions, requiring more time to maintain choice accuracy.

In addition, during testing, HA and LA did not differ in choice behavior to old pairs of stimuli, appearing to be able to retain what had been previously learned when behavior was no more reinforced. However, when faced by new pairs of stimuli, although groups showed comparable accuracy in choice behavior, the analysis on response times indicated a difficulty of HA in efficiently avoiding stimuli, which had acquired negative value, encountered in a new context, specifically when having to avoid the most negative stimulus among two negative stimuli. Indeed, in high-conflict choices, while LA were equally efficient in avoiding negative or choosing positive stimuli, HA were slower when accurately avoiding negative stimuli than when choosing positive ones and were also slower than LA. Therefore, HA appear less able to generalize the value of previously learned actions that specifically lead to a negative outcome to make adaptive choices in a new context, in particular when having to avoid the worst of two evils. Furthermore, performance in the testing phase is also informative about the quality of previous learning and enables to determine whether participants learn more from reward or punishment (Maia and Frank, 2011). In HA, the worse performance in the avoidance of negative stimuli relative to choice of positive stimuli suggests that the sensitivity to learn specifically from punishment, rather than reward, is impaired in alexithymia. Finally, this difficulty was found to increase with increasing tendency of individuals to have an externally oriented thinking style, more focused on concrete aspects of life and avoidant of introspection and affective thinking. Interestingly, externally oriented thinking in alexithymia has also been associated with an impoverished fantasy life and imaginative capacity (Sifneos, 1973; Taylor et al., 1991), two aspects that are crucially dependent on the use of the internal representations acquired through past learning (Hassabis et al., 2007; Schacter et al., 2007; Bertossi et al., 2016). Generally, the difficulty in identifying and describing feelings are seen as the core deficits in alexithymia, rather than the externally oriented thinking. However, the current results appear to shed new light on this alexithymia factor. Also, given the multifaceted nature of the difficulties in emotional processing of alexithymia multiple mechanisms may be core to this subclinical trait. In particular, while the interoception and emotional embodiment accounts of alexithymia, described in the introduction, may be related to the difficulties in identifying and describing feelings, impaired emotional learning may be more closely related to externally oriented thinking.

Overall, the current results indicate that individuals with alexithymia show impaired ability to learn the value of aversively motivated actions. In particular, although during the learning phase of the task HA were able to terminate aversively motivated actions, during the testing phase, when stimuli were encountered in a new context, HA needed more time to avoid the most negative stimulus among two negative stimuli. This result extends our previous findings, showing that alexithymia hinders learning about the aversive value of conditioned stimuli during Pavlovian learning (Starita et al., 2016), to instrumental learning, showing that a similar impairment is found also when learning the value of one’s own actions. Taken together, these results offer the opportunity for a new understanding of alexithymia as impairment in constructing the internal representations of emotional stimuli and actions, and in particular negatively valenced ones. In fact, Pavlovian and instrumental learning are two crucial processes through which previously neutral stimuli and actions acquire emotional value by being associated with aversive or appetitive stimuli, which are biologically prepared to trigger an emotional response (Pavlov, 1927; LeDoux, 1998; Daw and Tobler, 2014). Therefore, the impairments in emotional learning of individuals with alexithymia suggest that alexithymia is characterized by an impaired ability to update the value of stimuli and actions, in order to construct internal representations that include, alongside stimuli and actions biologically prepared to elicit an emotional response, those that are associated with them. Crucially, given the predictive nature of such representations (Öhman and Mineka, 2001; McNally and Westbrook, 2006; den Ouden et al., 2012) and consequently their fundamental role for effective processing of emotional stimuli (Bubic et al., 2010), the impaired construction of internal representations of emotional stimuli and actions in alexithymia may represent a mechanism that can account for their difficulties in emotional processing, especially for negatively valenced stimuli.

The selective impairment in learning from punishment, which manifested in high-conflict conditions, is also in line with the broader literature on alexithymia, which reports that difficulties in emotion processing may be more pronounced for negatively than positively valenced stimuli. For example, individuals with alexithymia rate the expression of fearful – but not happy – faces as less intense (Prkachin et al., 2009), fail to show enhanced remapping of fear on their own somatosensory, while having preserved remapping of happiness (Scarpazza et al., 2014, 2015) and fail to show enhanced electrophysiological response to fearful body postures, while having preserved response to happy ones (Borhani et al., 2016). Furthermore, the evidence of the impairment in the high-conflict condition highlights the subclinical nature of alexithymia, suggesting that difficulties in emotion processing may become evident only under high task demands and may not necessarily be evident in everyday life. This is in line, for example, with the findings on the difficulties of alexithymic individuals in the identification of emotional facial expressions, which are evident when stimuli are presented under temporal constraints (e.g., 66 or 100 ms) but not when stimulus exposure time is prolonged (e.g., 1 or 3 s) (Pandey and Mandal, 1997; Grynberg et al., 2012; Ihme et al., 2014a,b; Starita et al., 2018).

Although we did not collect data on the neural response during task completion, we wish to propose an interpretation of the results that also considers the possible neural mechanisms underlying the observed group differences. In fact, individuals’ performance on the PST has been previously related to variations in the error related negativity (ERN) event related potential and in activity of the dopaminergic system, which is in line with the role of these mechanisms in diving reinforcement learning (Sutton and Barto, 1998). For example, a previous study on the general population found that participant who learned more from reward than punishments had smaller ERN than participants who learned more from punishments than reward (Frank et al., 2005). Therefore, it is possible that the reduced ability of HA to learn from punishments may be accompanied by reduced ERN, when compared to LA. In keeping with this hypothesis, a study found that HA failed to exhibit enhanced ERN in an emotional (vs. neutral) Stroop task compared to LA (Maier et al., 2016). Additionally, the ability to avoid negative stimuli, in particular, seems to be related to differences in genotype associated to density of postsynaptic D2 receptors, which are crucial for learning from low dopamine levels, as in the case of dopamine dips following negative feedback (Frank et al., 2004; Maia and Frank, 2011). Indeed, performance in avoiding negative stimuli increases with increasing density of D2 receptors (Frank et al., 2007), and decreases in individuals carrying an allele of a genetic polymorphism associated with a reduction in D2 receptor density by up to 30% (Klein et al., 2007). Therefore, it might be possible that alexithymia may be related to differences in the dopamine system and in particular in those aspects supporting learning from negative feedback. In this regard, one study found that carriers of an allele associated with a reduction in D2 dopamine receptor, together with an allele associated with lower activity-dependent secretion of brain-derived neurotrophic factor, had significantly higher scores of alexithymia, compared to participants with other allelic variations (Klein et al., 2007). Therefore, it is possible that differences in functioning of the dopamine system may underlie the impairment in constructing the internal representations of emotional stimuli, specifically negative ones, in alexithymia. Future neuroimaging studies could empirically test such hypothesis.

Interestingly, impairments in emotional learning have been reported in a number of clinical conditions, such as in individuals with anxiety, depression and substance abuse (Gradin et al., 2011; Heinz et al., 2016; Keramati et al., 2017; White et al., 2017; Kumar et al., 2008, 2018). Additionally, epidemiological studies have found higher prevalence of alexithymia in these clinical populations compared to healthy controls (Berthoz et al., 1999; Honkalampi et al., 2000; Marchesi et al., 2000; Farges et al., 2004; Kun and Demetrovics, 2010; Panaite and Bylsma, 2012; Li et al., 2015; Craparo et al., 2016). This raises the possibility that alexithymia and its impaired emotional learning might represent a transdiagnostic factor across such pathologies.

## Conclusion

To conclude, the results of the current study indicate that alexithymia is related to an impairment in learning the value of aversively motivated actions during instrumental learning. Therefore, individuals with alexithymia may be unable to construct internal representations of emotional events that include not only stimuli and actions biologically prepared to elicit an emotional response but also those that are associated with them. Unable to predict the emotional future without such representations, individuals with alexithymia may be at the mercy of emotional stimuli, especially negative ones. Rather than prepare their response in advance, individuals with alexithymia can only respond to emotional stimuli once they have already occurred, hindering effective recognition, response and response regulation to emotional stimuli. Therefore, the impaired construction of internal representations of emotional stimuli and actions in alexithymia may represent a possible mechanism that can account for their multifaceted difficulties in emotional processing.

## Data Availability Statement

The raw data supporting the conclusions of this manuscript will be made available by the authors, without undue reservation, to any qualified researcher.

## Author Contributions

FS and GdP conceived and designed the study. FS acquired and analyzed the data and drafted the manuscript. GdP critically revised the manuscript for important intellectual content.

## Conflict of Interest Statement

The authors declare that the research was conducted in the absence of any commercial or financial relationships that could be construed as a potential conflict of interest.
